# Supplementary material for: The diamine cation is not a chemical example where density functional theory fails
Source: Nat Commun. 2018 Nov 9;9:4733. doi: 10.1038/s41467-018-07266-y (PMC6226436; doi:10.1038/s41467-018-07266-y)
Supplement: Supplementary file 1 — Supplementary Information [file 41467_2018_7266_MOESM1_ESM.pdf]

Supplementary Information for "The Diamine Cation Is Not a Chemical Example Where Density Functional Theory Fails"

*Zulfikhar A. Ali[1], Fredy W. Aquino[2], and Bryan M. Wong[1,2]**

[1]Department of Physics & Astronomy, University of California-Riverside, Riverside, CA 92521, United States

[2]Department of Chemical & Environmental Engineering and Materials Science & Engineering Program, University of California-Riverside, Riverside, CA 92521, United States

*Corresponding author. E-mail: bryan.wong@ucr.edu. Homepage: http://www.bmwong-group.com

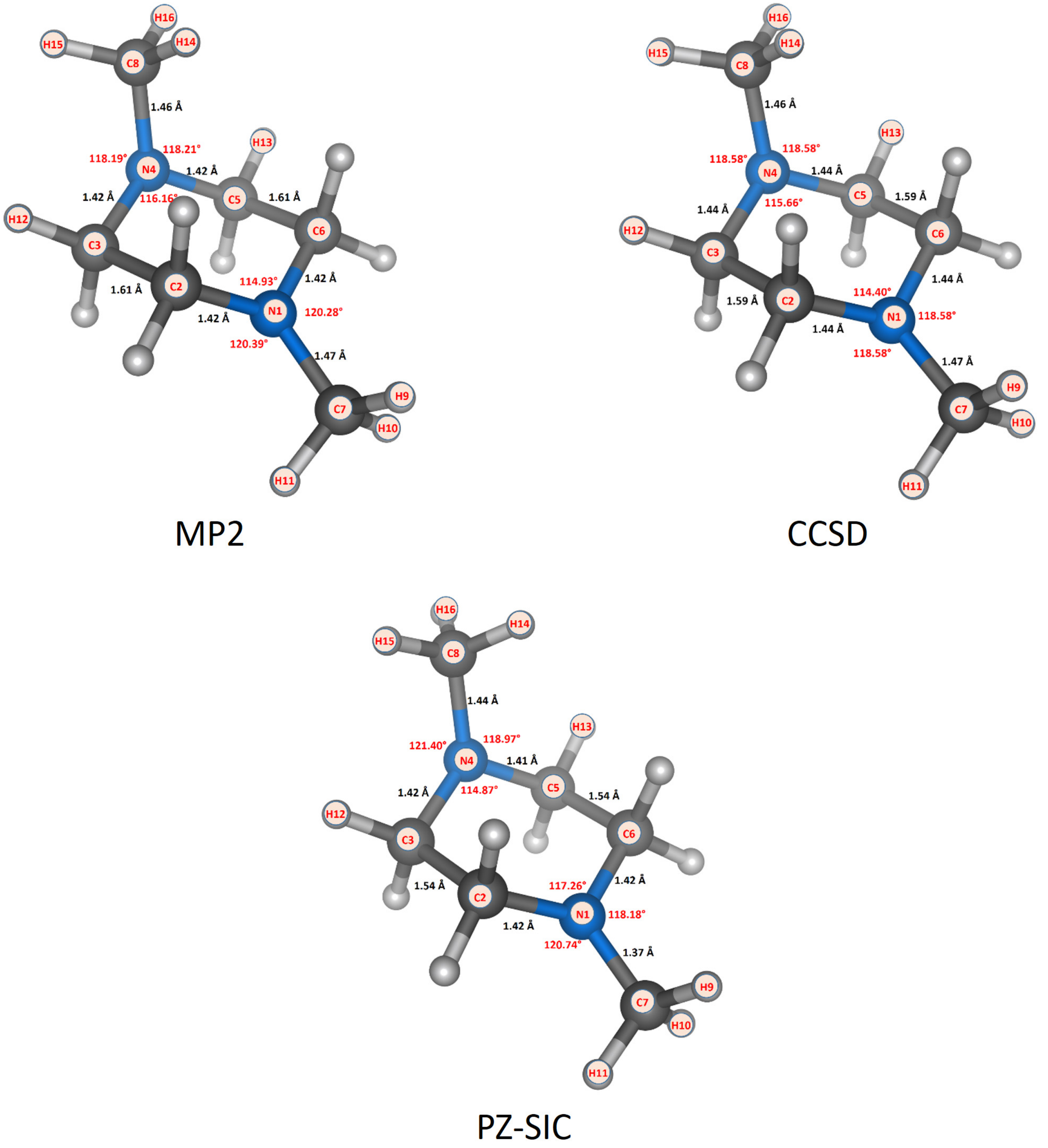

Supplementary Fig. 1. Comparison of transition-state geometries for the diamine cation optimized with the MP2, CCSD, and PZ-SIC computational methods.

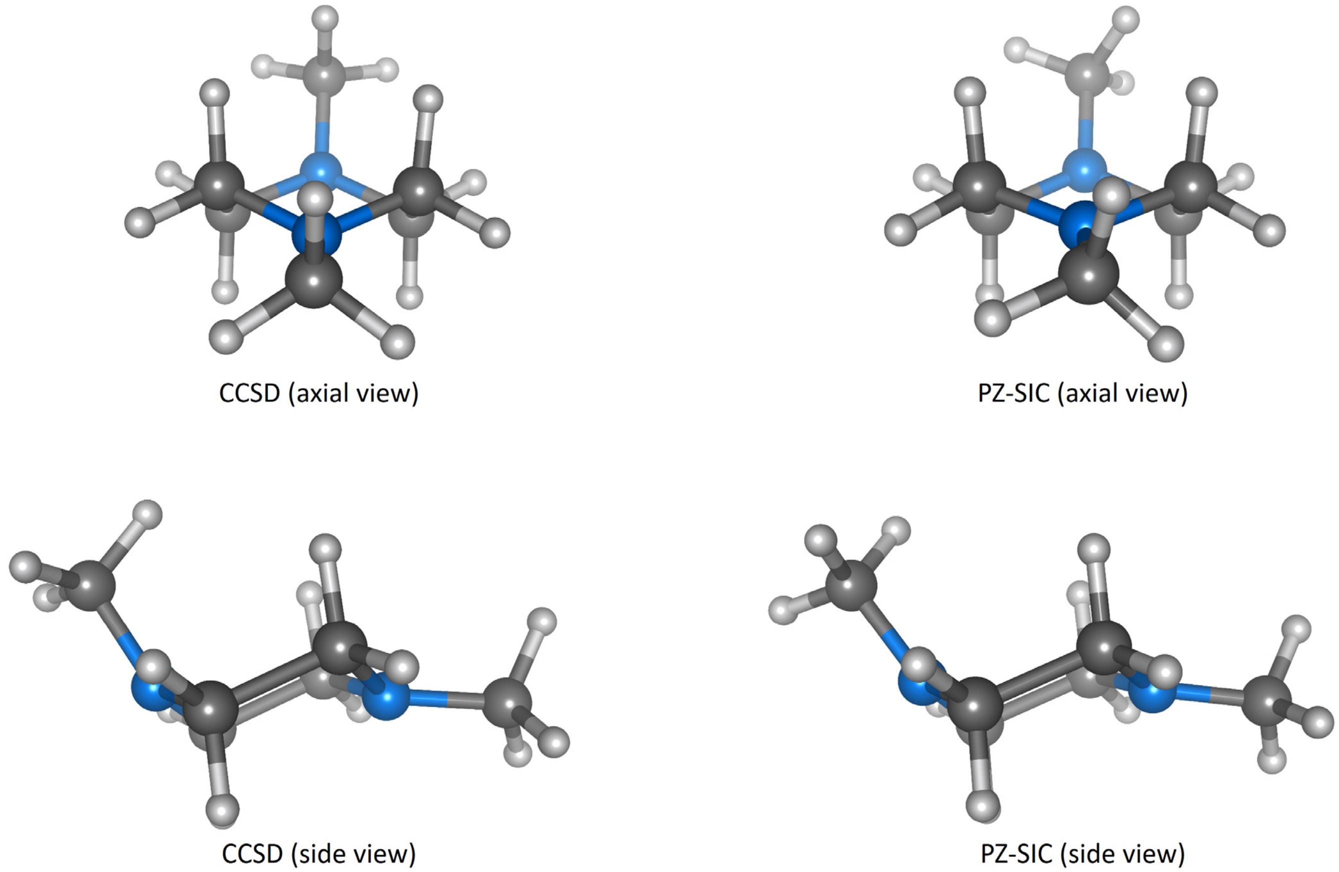

Supplementary Fig. 2. Axial and side views of transition-state geometries for the diamine cation optimized with the CCSD and PZ-SIC computational methods.

Supplementary Table 1: Comparison of various geometric parameters for the diamine cation transition state obtained with the MP2, CCSD, and PZ-SIC computational methods. The atom labels and numbers refer to the labeling scheme used in **Supplementary Figure 1**. The shaded entries denote geometric parameters with the largest variation between the MP2/CCSD and PZ-SIC approaches.

	**Bond Length (Å)**		
	**MP2**	**CCSD**	**PZ-SIC**
C7-N1	1.47	1.47	1.37
N1-C2	1.42	1.44	1.42
C2-C3	1.61	1.59	1.54
C3-N4	1.42	1.44	1.42
N4-C5	1.42	1.44	1.41
C5-C6	1.61	1.59	1.54
C6-N1	1.42	1.44	1.42
N4-C8	1.45	1.46	1.44

	**Bond Angle (degrees)**		
	**MP2**	**CCSD**	**PZ-SIC**
C7-N1-C2	120.39	118.58	120.74
C7-N1-C6	120.28	118.58	118.11
C8-N4-C3	118.19	118.58	121.40
C8-N4-C5	118.21	118.58	118.97
C3-N4-C5	116.16	115.66	114.87
C2-N1-C6	114.93	114.40	117.26

	**Dihedral Angle (degrees)**		
	**MP2**	**CCSD**	**PZ-SIC**
C3-C2-N1-C7	-141.54	-150.91	-148.89
C2-C3-N4-C8	-86.80	-89.82	-94.33
C6-C5-N4-C8	86.79	89.82	94.75
C5-C6-N1-C7	141.53	150.91	148.51
C2-N1-C7-H9	-77.83	-73.17	-90.63
C2-N1-C7-H10	161.31	166.14	151.76
C2-N1-C7-H11	43.03	47.52	28.90
C6-N1-C7-H9	77.73	73.18	66.55
C6-N1-C7-H10	-43.13	-47.50	-51.06
C6-N1-C7-H11	-161.42	-166.12	-173.91
C3-N4-C8-H14	74.26	74.53	107.13
C3-N4-C8-H15	-45.07	-44.92	-12.45
C3-N4-C8-H16	-166.40	-166.02	-133.79
C5-N4-C8-H14	-74.51	-74.61	-47.18
C5-N4-C8-H15	166.16	165.94	-166.77
C5-N4-C8-H16	44.84	44.84	71.89
N1-C2-C3-H12	-175.99	-175.69	-173.57
N1-C6-C5-H13	176.03	175.70	172.39

Supplementary Table 2: Reference Cartesian coordinates (in Å) for the MP2-optimized DMP-L^+ molecular cation.

N	0.8669040000	1.0704380000	0.0000000000
C	0.1612010000	0.6287860000	1.1938500000
C	0.1612010000	-0.9247560000	1.2290130000
N	-0.4616440000	-1.3789290000	0.0000000000
C	0.1612010000	-0.9247560000	-1.2290130000
C	0.1612010000	0.6287860000	-1.1938500000
C	1.0464460000	2.5339080000	0.0000000000
C	-1.8315630000	-1.8536180000	0.0000000000
H	-0.8886810000	0.9965970000	1.2408420000
H	0.6910660000	0.9796830000	2.0924050000
H	-0.3948770000	-1.3161980000	2.0904220000
H	1.2045650000	-1.2762920000	1.2365510000
H	0.6910660000	0.9796830000	-2.0924050000
H	-0.8886810000	0.9965970000	-1.2408420000
H	1.2045650000	-1.2762920000	-1.2365510000
H	-0.3948770000	-1.3161980000	-2.0904220000
H	1.6186930000	2.8236570000	0.8921360000
H	0.0779740000	3.0737480000	0.0000000000
H	1.6186930000	2.8236570000	-0.8921360000
H	-2.0156980000	-2.4438030000	-0.9065480000
H	-2.5030430000	-0.9717060000	0.0000000000
H	-2.0156980000	-2.4438030000	0.9065480000

Supplementary Table 3: Reference Cartesian coordinates (in Å) for the MP2-optimized DMP-D^+ molecular cation.

```
N -0.5345895389  1.3087803821 -0.0000000006
C -0.6076389847  0.5258335438  1.1961899695
C  0.6076396600 -0.5258341301  1.1961895184
N  0.5345917563 -1.3087816692 -0.0000000008
C  0.6076396610 -0.5258341309 -1.1961895206
C -0.6076389837  0.5258335428 -1.1961899716
C  0.3752547937  2.4554774414  0.0000000013
C -0.3752553891 -2.4554764973  0.0000000006
H -1.5467752809 -0.0494318097  1.2197931686
H -0.5248208759  1.1610168822  2.0895997652
H  0.5248206257 -1.1610168488  2.0895996511
H  1.5467759018  0.0494313515  1.2197940032
H -0.5248208747  1.1610168827 -2.0895997661
H -1.5467752807 -0.0494318095 -1.2197931687
H  1.5467759027  0.0494313505 -1.2197940087
H  0.5248206252 -1.1610168511 -2.0895996516
H  0.1877028870  3.0620743686  0.8958603262
H  0.1877028889  3.0620743723 -0.8958603212
H  1.4356271683  2.1334602394  0.0000000015
H -0.1877045140 -3.0620740720 -0.8958601190
H -1.4356276341 -2.1334584711  0.0000000005
H -0.1877045138 -3.0620740676  0.8958601228
```

Supplementary Table 4: Reference Cartesian coordinates (in Å) for the MP2-optimized transition state of the diamine molecular cation.

N	0.5169376764	1.1624721594	-0.0043853521
C	-0.0451819170	0.6525896513	1.1954852164
C	0.2162493170	-0.9379387657	1.2083443371
N	-0.3585603333	-1.4297639692	0.0041178155
C	0.2090314973	-0.9437533629	-1.2055308074
C	-0.0528041300	0.6474273750	-1.1981536662
C	1.2053913627	2.4578724505	-0.0095705479
C	-1.7689762346	-1.7788119017	0.0093772545
H	-1.1434665994	0.7816116871	1.2867889588
H	0.4504144731	1.0826153945	2.0777503663
H	-0.2657739082	-1.3862848409	2.0870216334
H	1.3034248099	-1.0878007401	1.2161733426
H	0.4369808769	1.0733476541	-2.0856411190
H	-1.1517443039	0.7752599973	-1.2828594262
H	1.2962575979	-1.0927655848	-1.2191034966
H	-0.2780782203	-1.3957409619	-2.0794962237
H	1.8456444047	2.5244848737	0.8812971522
H	0.5007534668	3.3079744178	-0.0101019721
H	1.8413753440	2.5197755019	-0.9038513631
H	-2.0076296657	-2.3591927147	-0.8907664415
H	-2.3812719858	-0.8530646307	0.0113746009
H	-2.0010665285	-2.3584946900	0.9117297381

Supplementary Table 5: Reference Cartesian coordinates (in Å) for the CCSD-optimized DMP-L^+ molecular cation.

```
N  0.8492470000  1.0785750000  0.0000000000
C  0.1517520000  0.6387710000  1.1999580000
C  0.1517520000 -0.9190230000  1.2370970000
N -0.4671290000 -1.3826540000  0.0000000000
C  0.1517520000 -0.9190230000 -1.2370970000
C  0.1517520000  0.6387710000 -1.1999580000
C  1.0988920000  2.5319720000  0.0000000000
C -1.8380220000 -1.8789370000  0.0000000000
H -0.9001810000  0.9999440000  1.2543460000
H  0.6867250000  0.9953650000  2.0940890000
H -0.4120010000 -1.3090020000  2.0956900000
H  1.1937630000 -1.2766870000  1.2561590000
H  0.6867250000  0.9953650000 -2.0940890000
H -0.9001810000  0.9999440000 -1.2543460000
H  1.1937630000 -1.2766870000 -1.2561590000
H -0.4120010000 -1.3090020000 -2.0956900000
H  1.6855120000  2.7954320000  0.8933120000
H  0.1578220000  3.1203250000  0.0000000000
H  1.6855120000  2.7954320000 -0.8933120000
H -2.0115770000 -2.4750280000 -0.9068480000
H -2.5243930000 -1.0070060000  0.0000000000
H -2.0115770000 -2.4750280000  0.9068480000
```

Supplementary Table 6: Reference Cartesian coordinates (in Å) for the CCSD-optimized DMP-D$^+$ molecular cation.

```
N  0.2368380000  1.3948160000  0.0000000000
C -0.2368380000  0.7727630000  1.1975510000
C  0.2368380000 -0.7727630000  1.1975510000
N -0.2368380000 -1.3948160000  0.0000000000
C  0.2368380000 -0.7727630000 -1.1975510000
C -0.2368380000  0.7727630000 -1.1975510000
C  1.6067020000  1.9205960000  0.0000000000
C -1.6067020000 -1.9205960000  0.0000000000
H -1.3384270000  0.7822810000  1.2287040000
H  0.1733680000  1.2649960000  2.0912800000
H -0.1733680000 -1.2649960000  2.0912800000
H  1.3384270000 -0.7822810000  1.2287040000
H  0.1733680000  1.2649960000 -2.0912800000
H -1.3384270000  0.7822810000 -1.2287040000
H  1.3384270000 -0.7822810000 -1.2287040000
H -0.1733680000 -1.2649960000 -2.0912800000
H  1.7548980000  2.5393440000  0.8966320000
H  1.7548980000  2.5393440000 -0.8966320000
H  2.3550490000  1.1017830000  0.0000000000
H -1.7548980000 -2.5393440000  0.8966320000
H -1.7548980000 -2.5393440000 -0.8966320000
H -2.3550490000 -1.1017830000  0.0000000000
```

Supplementary Table 7: Reference Cartesian coordinates (in Å) for the CCSD-optimized transition state of the diamine molecular cation.

```
N  0.6158602154  1.1338056095 -0.0078384890
C  0.0222013504  0.6417989195  1.2037815406
C  0.2031723820 -0.9330104953  1.2273619047
N -0.3867270858 -1.4374022491  0.0089457548
C  0.1958489089 -0.9461387102 -1.2183074413
C  0.0149043271  0.6288482298 -1.2104989320
C  1.1698597856  2.4929227602 -0.0167553229
C -1.8022556033 -1.7984516066  0.0151541513
H -1.0667362027  0.8469400541  1.2847998802
H  0.5339765420  1.0688375637  2.0794892256
H -0.3034891298 -1.3669072394  2.1008794022
H  1.2788744902 -1.1605953845  1.2438856424
H  0.5213270809  1.0464709553 -2.0938243751
H -1.0745253827  0.8330695380 -1.2871295679
H  1.2714498571 -1.1738621298 -1.2387681730
H -0.3159679063 -1.3893957778 -2.0840856952
H  1.7999951737  2.6294023607  0.8755362601
H  0.3769644107  3.2657865968 -0.0183697606
H  1.7943719501  2.6199133624 -0.9143821126
H -2.0339073493 -2.3868172270 -0.8837006922
H -2.4191921027 -0.8750508197  0.0128501283
H -2.0280987115 -2.3783453106  0.9209766715
```
